# Supplementary material for: Fabrication and microfluidic analysis of graphene-based molecular communication receiver for Internet of Nano Things (IoNT)
Source: Sci Rep. 2021 Oct 1;11:19600. doi: 10.1038/s41598-021-98609-1 (PMC8486847; doi:10.1038/s41598-021-98609-1)
Supplement: Supplementary file 1 — Supplementary Information. [file 41598_2021_98609_MOESM1_ESM.pdf]

Fabrication and Microfluidic Analysis of Graphene-based Molecular Communication Receiver for Internet of Nano Things (IoNT)

Murat Kuscu[1,2,3*], Hamideh Ramezani[1,2], Ergin Dinc[1,4], Shahab Akhavan[2,5], and Ozgur B. Akan[1,3]

[1]Internet of Everything (IoE) Group, Department of Engineering, University of Cambridge, Cambridge, CB3 0FA, UK
[2]Cambridge Graphene Centre (CGC), Department of Engineering, University of Cambridge, Cambridge, CB3 0FA, UK
[3]Department of Electrical and Electronics Engineering, Koc University, Istanbul, 34450, Turkey
[4]Battcock Centre for Experimental Astrophysics, Cavendish Laboratory, University of Cambridge, Cambridge, CB3 0HE, UK
[5]Institute for Materials Discovery, University College London, Torrington Place, London WC1E 7JE, UK
[*]mk959@cantab.ac.uk

1. Mobility Analysis

The mobility of the GFET channels is calculated as (240.62 ± 23.47) cm^2/V·s using the formula:

$$\mu = \frac{L}{W}\frac{t_{ox}}{\varepsilon_{ox}}\frac{|g_m|}{V_{ds}} \tag{1}$$

where $g_m = \partial I_{ds}/\partial V_{bg}$ is the transconductance of the GFET channels with I_{ds} and V_{bg} being the drain-to-source current and the back-gate voltage, respectively. $L = 160$ μm and $W = 100$ μm are the length and the width of the GFET channels, respectively. $t_{ox} = 90$ nm and $\varepsilon_{ox} = 3.45 \times 10^{-14}$ F/cm are the thickness and the permittivity of the SiO_2 layer, respectively. $V_{ds} = 100$ mV is the drain-to-source voltage.

The transconductance of the GFET channels g_m is obtained using the linear approximation of the transfer curve in the hole-carrier region. The transfer characteristics, shown in Supplementary Figure 1 for four channels of the MC receiver, are obtained in a back-gate configuration before functionalisation of the GFET channels.

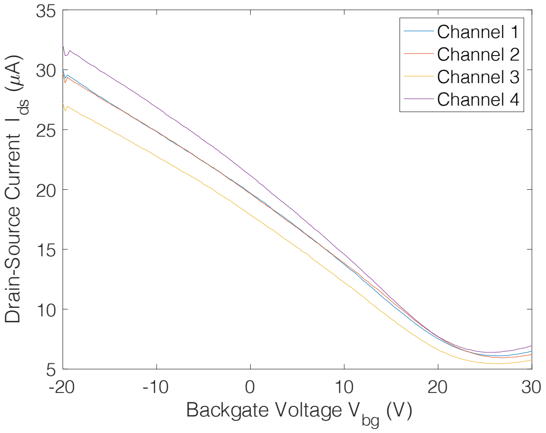

Figure 1. Transfer characteristics of four channels obtained in back-gate configuration with the back-gate voltage V_{bg} being swept between -20 V and 30 V in the forward direction and $V_{ds} = 100$ mV.

2. Hysteresis Analysis

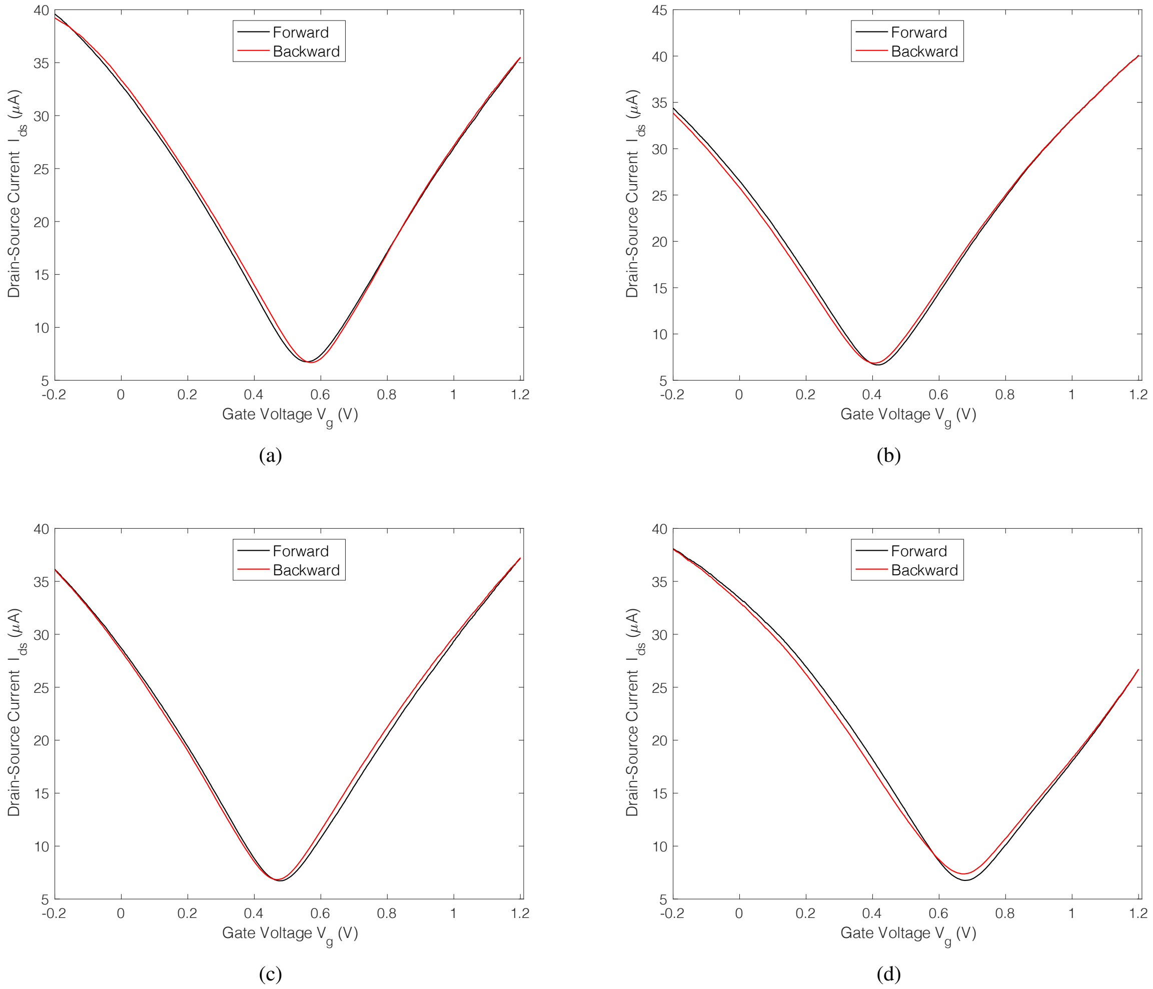

Figure 2. Hysteresis analysis of the MC receiver: Drain-source current I_{ds} with forward and backward sweep of gate voltage V_g at 140 mV/s sweep rate. (a) Before functionalisation. (b) After functionalisation with PBASE. (c) After immobilization of pDNA. (d) After passivation with ethanolamine.

3. Leakage Current Analysis

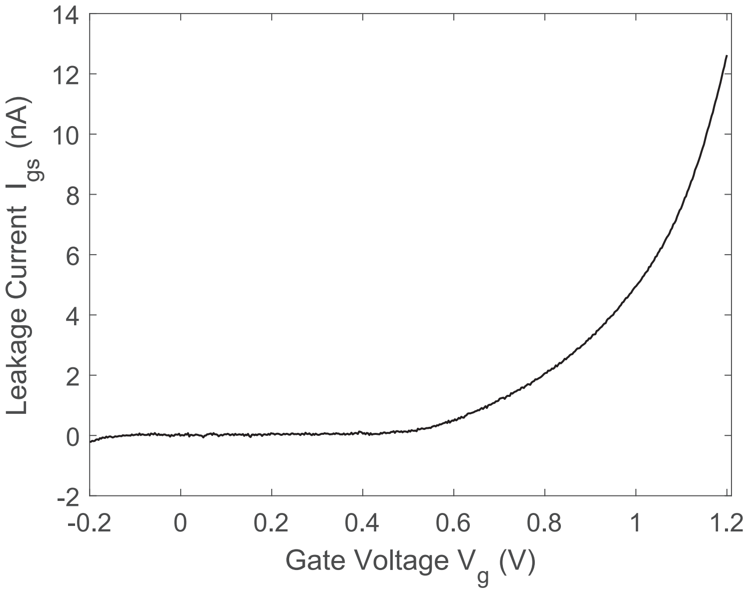

Figure 3. Leakage current analysis of the MC receiver: Gate-source current I_{gs} with varying gate voltage V_g.

4. Computer-Aided Design for the Optical Lithography of the MC Receiver

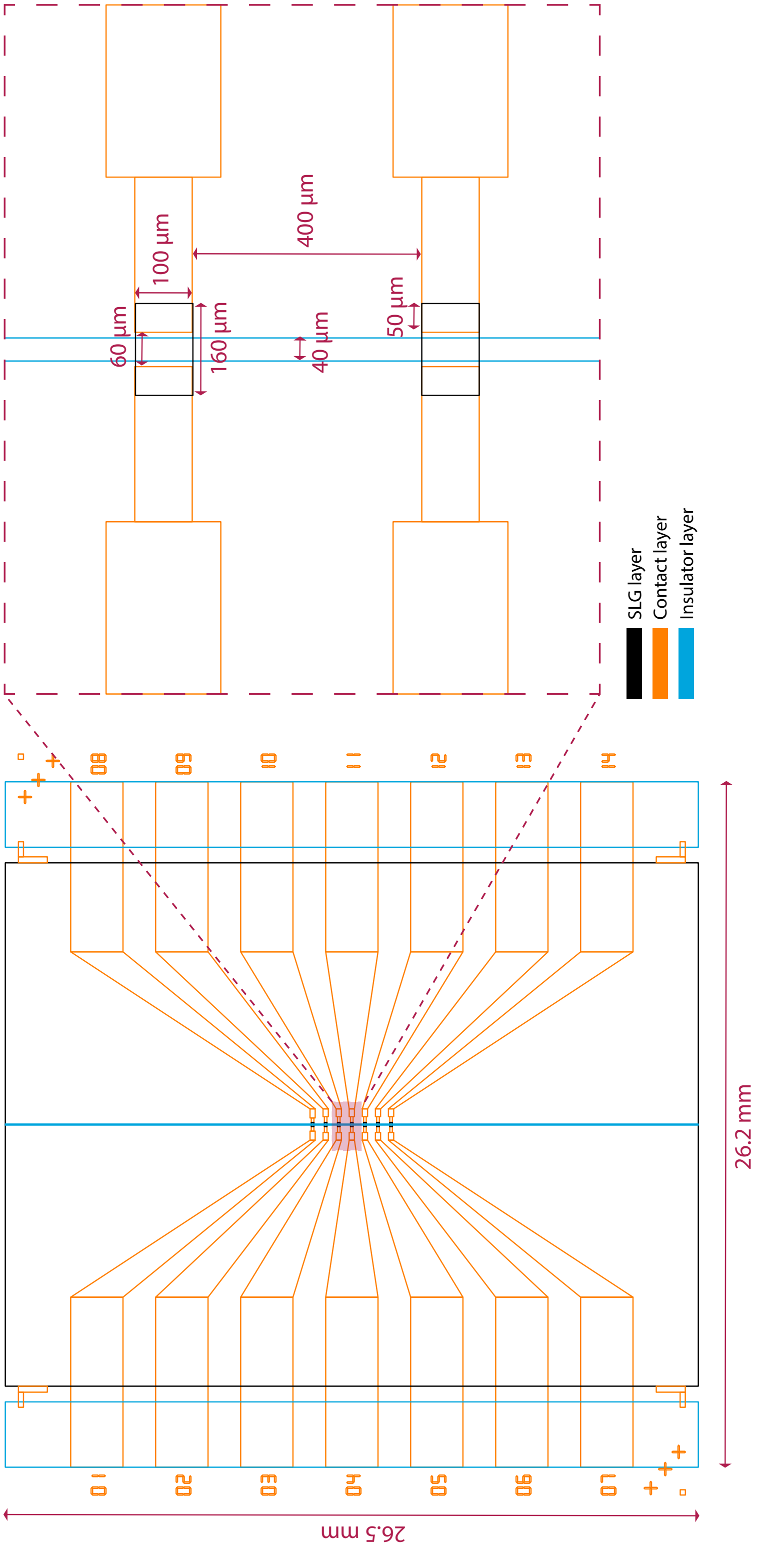

Figure 4. Computer-aided design for optical lithography of MC receiver.

5. Computer-Aided Design for the 3d Printing of PDMS Mould

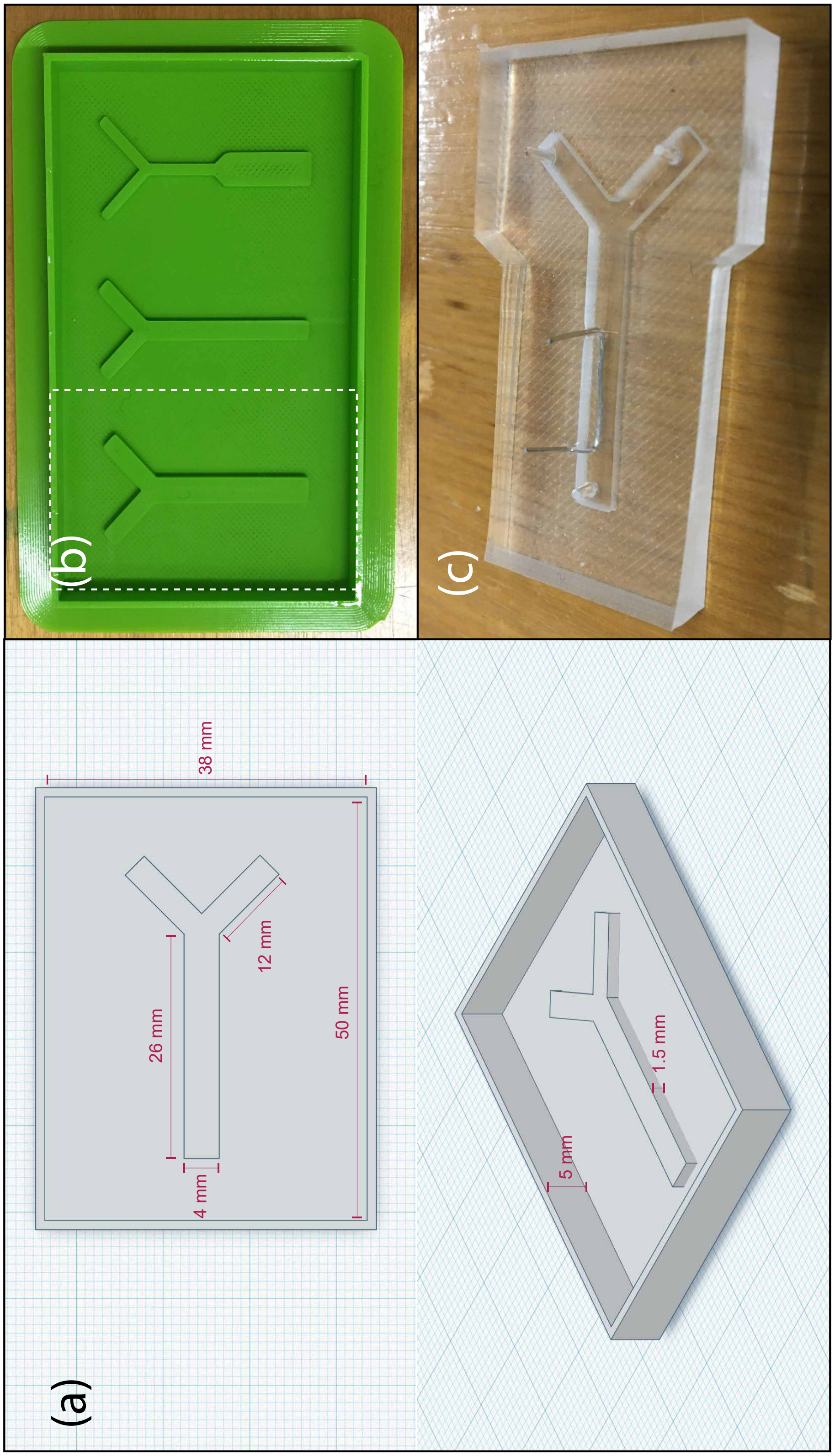

Figure 5. (a) Computer-aided design for 3d printing of PDMS mould. (b) 3d-printed PDMS mould. (c) PDMS microfluidic channel with the Pt wire mounted on top as the gate electrode.
